# Recapitulating influenza virus infection and facilitating antiviral and neuroprotective screening in tractable brain organoids

**DOI:** 10.7150/thno.75123

**Published:** 2022-07-07

**Authors:** Xiaodong Zhang, Haishuang Lin, Liangzhen Dong, Qing Xia

**Affiliations:** 1 Department of Chemical Biology, State Key Laboratory of Natural and Biomimetic Drugs, School of Pharmaceutical Sciences, Peking University, Beijing, China.; 2 Institute of Laboratory Animal Science, Chinese Academy of Medical Sciences and Comparative Medicine Center, Peking Union Medical College, Beijing, China.

**Keywords:** Influenza virus, Brain organoids, Human pluripotent stem cells, RNA transcriptomic profiling, Antiviral screening.

## Abstract

Human pluripotent stem cell derived brain organoids offer an unprecedented opportunity for various applications as *in vitro* model. Currently, human brain organoids as models have been used to understand virus-induced neurotoxicity.

**Methods:** The brain organoids were separately challenged by multiple viruses including influenza viruses (H1N1-WSN and H3N2-HKT68), Enteroviruses (EV68 and EV71) and Severe Fever with Thrombocytopenia Syndrome Virus (SFTSV) to investigate the impaired effect of these viruses on human brain development.

**Results:** The brain organoids challenged by influenza viruses had decreased overall organoid size, while enteroviruses infected brain organoids displayed the opposite result. Then, we found WSN preferentially infected MAP2+ neurons compared to SOX2+ neural stem cells (NSCs) and GFAP+ astrocytes in brain organoids, and induced apoptosis of NSCs and neurons, and released inflammatory factors (TNF-α, INF-γ, and IL-6), facilitating brain damage. Furthermore, transcriptional profiling revealed several co-upregulated genes (*CSAG3* and *OAS2*) and co-downregulated genes (*CDC20B*, *KCNJ13*, *OTX2-AS1*) after WSN infection for 24 hpi and 96 hpi, implicating target for antiviral drugs development. Finally, we explored compound PYC-12 could significantly suppress virus infection, apoptosis, and inflammatory responses.

**Conclusions:** Collectively, we established a tractable experimental model to investigate the impact and mechanism of virus infection on human brain development.

## Introduction

Central nervous system (CNS) infections are one of the most critical public health problems due to its high morbidity and mortality every year, predominately in young children, the elderly, and the immunocompromised person 1-3, and often accompany with neurological sequelae, including epilepsy 4, and neurodevelopmental disorders, such as attention-deficit/hyperactivity disorder (ADHD) 5 and autism spectrum disorder (ASD) 6. Some known neurotropic viruses, such as measles virus (MV), herpes virus, and human immunodeficiency virus (HIV), can cause CNS infections 7. Moreover, respiratory viruses including human respiratory syncytial virus (HRSV), influenza virus (IV), and coronavirus (Cov) have also become key factors responsible for CNS pathologies. In particular, COVID-19, which is caused by severe acute respiratory syndrome coronavirus 2 (SARS-CoV-2), has been characterized by respiratory failure in critically ill patients. The outbreak of COVID-19 has led to a pandemic, and serious and even fatal manifestations have been seen in the brain 8. The annual pandemic of influenza A virus (IAV) has been shown to cause neurodegenerative diseases in clinical investigations 9. IAV is often the causative agent of upper respiratory infection by recognizing sialic acids (SA) receptor in humans affecting people of all ages 10. Although the influenza virus primarily infects the lungs, its neuropathological effects have also been shown in the clinic, including febrile seizures, myelitis, focal encephalitis, and even meningitis 11. However, the neuropathological mechanism of IAV remains elusive mainly due to the lack of *in vitro* physiological models instead of patient derived brain tissue.

Human pluripotent stem cell (hPSC)-derived organoids, which are an *in vitro* self-organizing and self-renewing three-dimensional tissue recapitulating the cytoarchitecture and functional components of *in vivo* human tissue, which exhibits significant advantages over cell lines and animal models in terms of tissue development, disease modeling, drug screening, and therapeutic discovery 12-15. For example, Chen *et al.* 16 generated serum-exposed brain organoids modeling Alzheimer's disease (AD)-like pathologies, which provide a powerful platform for both mechanistic study and therapeutic development of AD treatments. Dang *et al.* 17 utilized human cerebral organoids to demonstrate that Zika virus (ZIKV) could decrease the number of neural progenitors through activation of the innate immune receptor TLR3, resulting in dysregulation of a network of genes involved in axon guidance, neurogenesis, differentiation, and apoptosis. In addition, there are a fair amount of studies on brain organoid infection with virus for neuropathogenesis or drug 15,16,18-24. Currently, several organoids (e.g., lung, liver, gut, brain, and kidney) mimicking different tissues have been used to study the effects of SARS-CoV-2 on tissues *in vitro*, and tissue histopathology 25. Eric *et al.* demonstrated that SARS-CoV-2 could replicate efficiently in human brain organoid with accompanying metabolic changes, and the brain of mice causing CNS-specific lethality 26. Although many evidences demonstrated the infection and persistence of influenza virus in animal brain and human brain 27-34, little evidence directly confirmed influenza virus replication in human brain or animal brain 35-37. Therefore, studying influenza virus-infected brain organoid to identify neuropathogenesis or for drug screening are necessary.

In this study, we evaluated the potential of brain organoids as *in vitro* infection models, and investigated the mechanism of neuropathogenesis underlying WSN infection. We found that WSN preferentially infected MAP2+ neurons in brain organoids, and further damaged the brain by eliciting inflammatory factor release (TNF-α, INF-γ, and IL-6), as well as inducing the apoptosis of NSCs and neurons, but not astrocytes. Additionally, RNA transcriptomic profiles revealed new protein targets and noncoding RNAs for the development of antiviral strategies. Finally, we conducted an antiviral study and found that some specific neurotrophic factors (e.g., BDNF, GDNF, and NT3) and a potential antiviral drug candidate, PYC-12, could both significantly decrease virus replication to achieve antiviral and neuroprotective effects. Collectively, human organoids were shown to serve as an invaluable tool in the field of virus research to investigate the molecular mechanisms underlying virus replication and antiviral drug screening.

## Methods

### Cell culture and reagents

Human HEK293T (CRL-11268) and Madin-Darby Canine Kidney (MDCK) cells (ATCC, CRL-2936) were maintained in Dulbecco's Minimal Essential Medium (DMEM) (Gibco) supplemented with 10% fetal bovine serum (Gibco), 100 units/mL penicillin, and 100 µg/mL streptomycin (Invitrogen). Authentication and test for the free of mycoplasma were performed with MycAway^TM^ one-step mycoplasma detection kit (Yeasen). Astrocytes were purchased from Cellapy (CA2315106) and cultured in NeuroEasy maintenance medium (Cellapy). Human embryonic stem cells (hESCs) were obtained from Harvard Stem Cell Institute. hESCs were routinely checked for pluripotent, normal karyotype, mycoplasma free and cultured in feeder-free conditions on Matrigel-coated plates with Essential 8 medium (GIBCO) and passaged with TrypLE^TM^ express (GIBCO). 10 μM Peramivir (M3222, AbMole BioScience), 1 μM Nucleozin (A3670, Apexbio), 50 μM PYC-12, 20 μM RO3306, 10 μM WH-L50B, 20 ng/mL BDNF (450-02, PeproTech), 20 ng/mL GDNF (450-10, PeproTech), 20 ng/mL NT3 (450-03, PeproTech) were used in this study.

### Viruses

Three H1N1 strains (WSN, CA07 and PR8), Enterovirus 68/71, and SFTSV obtained from Academy of Military Medical Sciences were used in this study. All viruses' stocks were prepared in MDCK cells and titrated by TCID_50_ on MDCK cells as described in details below. Studies with infectious H1N1 were conducted under biosafety level 2 (BSL-2) conditions at the Peking University Health Science Center with approval from Institutional Biosafety Committee.

### Neural stem cells (NSCs) differentiation

hESCs were differentiated into NSCs on Matrigel-coated plates using the monolayer protocol as previously described 38. Briefly, hESCs were first dissociated into single cells with Accutase (STEMCELL). Then, hESCs were plated onto Matrigel at a density of 2x10^5^ cells/cm^2^ in E8 medium containing 10 μM ROCK inhibitor (STEMCELL Technologies) and cultured overnight. 24 h later, cells were changed to E6 medium (GIBCO) containing 10 μM SB431542 (STEMCELL) and 100 nM LDN193189 (Selleckchem) to initiate differentiation. Medium was changed every day until day 7. Day 7-NSCs were used for the following experiments.

### Brain organoids generation and culture

Brain organoids were generated from hESCs as previously described 39, but slightly modified. Briefly, for embryoid body (EB) formation, hESCs were washed twice with DPBS, incubated with Accutase for 5 minutes, and dissociated into single cells. 3000 single cells were seeded in each well of low attachment 96-well U-bottom plate in E8 medium containing 10 μM ROCK inhibitor and centrifuged at 100 g for 3 min, then medium was half changed every other day. On day 4, EBs were transferred to low attachment 24-well plate in neural induction medium containing DMEM-F12 (GIBCO) with 1% N2 supplement (GIBCO), 1% Glutamax supplement (GIBCO), 1% MEM-NEAA (GIBCO) and 1 μg/mL Heparin (GIBCO), and medium was changed after 48 h. On day 7, EBs were transferred into Matrigel droplets as previously described and cultured in brain organoid differentiation media containing 50% DMEM-F12, 50% Neurobasal, 200x N2 supplement, 0.025% Insulin (GIBCO), 100x Glutamax supplement, 200x MEM-NEAA, 100x penicillin-streptomycin, 0.035% 2-Mercaptoethanol and 100x B27 supplement without Vitamin A, and medium was changed after 48 h. On day 10, organoids were transferred to orbital shaker (Corning) in brain organoid differentiation media with Vitamin A, medium was changed every 4 days.

### Virus infection

For cell line infection, H9, NSCs and astrocytes were seeded in chamber at 1x10^6^ cells. The cells were then rinsed with PBS, and WSN was diluted to the desired multiplicity of infection (MOI) of 1 according to dissociating the organoid and added to the cells. The cells were incubated for 2 h at 37 °C. The supernatant was removed and the cells were washed twice with PBS. Culture medium with 1% FBS and 1000x TPCK (final concentration 1 μg/mL) was added to each well, and cells were incubated at 37 °C and 5% CO_2_, after 24 h infection, cells were prepared for immunofluorescence staining. For brain organoids infection, organoids were transferred to low attachment 24-well plate and washed twice with DPBS, and WSN was diluted to the desired multiplicity of infection (MOI) of 1 and added to the cultured medium. The organoids were incubated for 8 h at 37 °C. The supernatant was removed and the cells were washed twice with DPBS, and then organoids were transferred to low attachment 6-well plate, 4 mL culture medium with 1% FBS and 1000x TPCK (final concentration 1 μg/mL) was added to each well, and organoids were incubated at 37 °C and 5% CO_2_ at a shake speed of 60 rpm, after 24 h and 96 h infection, organoids were prepared for immunofluorescence staining, RNA extraction and RNA-seq, and the supernatant was harvested for ELISA and virus titration.

### Immunofluorescence staining

For cell immunofluorescence staining, the cells were fixed with 4% PFA at room temperature for 10 min, permeated with PBST (PBS with 0.1% Triton X-100) for 20 min and blocked with 1% BSA for 30 min. Then the cells were incubated with primary antibodies listed in Table S1 at 4 °C overnight. The cells were subsequently incubated with secondary antibodies listed in Table S1 at room temperature for 1 h. The cells were mounted with mounting fluid containing DAPI (Yeason, 36308ES11). For organoid immunofluorescence staining, the organoids were fixed with 4% PFA at room temperature for 30 min, then immersed in 30% (w/v) sucrose until submersion before embedding and freezing in the Optimal Cutting Temperature (OCT) compound (Tissue-Tek). Serial 12 µm sections were obtained by cryo-sectioning of the embedded organoid at -20 °C using a cryostat (Leica). Cryosections were permeated with in PBST at temperature for 30 min and blocked with sheep serum (Zhongshanjinqiao, ZLI-9022) for 1 h. The sections were incubated with primary antibodies listed in Table S1 diluted in blocking buffer at 4 °C overnight. The slides were subsequently incubated with secondary antibody at room temperature for 1 h. The slides were mounted with mounting fluid containing DAPI. Stained sections were photographed under a Nikon Ti-S microscope. Apoptotic cells were labelled with Click-It Plus TUNEL assay (C10619, ThermoFisher Scientific).

### RNA isolation and quantitative RT-PCR

Total RNA was extracted from cells or organoids using the Quick-RNA MicroPrep kit (Zymo Research). RNA was subjected to quantitative real-time PCR in accordance with the protocol provided by one-step SYBR green RT-PCR Kit (Cwbio). The transcripts were quantitated and normalized to the internal GAPDH control. The primers used in the experiments are listed in **Table S2.** The PCR conditions were 1 cycle at 95 °C for 5 min, followed by 40 cycles at 95 °C for 15 s, 60 °C for 1 min, and 1 cycle at 95 °C for 15 s, 60 °C for 15 s, 95 °C for 15 s. The results were calculated using the 2^-△△CT^ method according to the GoTaq qPCR Master Mix (Promega) manufacturer's specifications.

### Virus quantification by 50% tissue culture infective dose

For quantifying all viruses' stocks, the 50% tissue culture infectious dose (TCID_50_/mL) titers were determined. In brief, 5×10^4^ HEK293T cells were seeded in 96-well plates the day before infection. The virus samples were serially diluted with DMEM containing 1% FBS (10^3^ to 10^10^) and then each of dilution was added in wells separately. The plates were incubated at 37 °C in 5% CO_2_ for 2-5 days. The cytopathic effect (CPE) was observed under a microscope and determined virus titer using the Reed-Münch endpoint calculation method.

### Microelectrode arrays (MEA)

Day 40 brain organoids were seeded onto 48-well transparent MEA plates. Brain organoids were cultured in brain organoid differentiation media containing 50% DMEM-F12, 50% Neurobasal, 200x N2 supplement, 0.025% Insulin (GIBCO), 100x Glutamax supplement, 200x MEM-NEAA, 100x penicillin-streptomycin, 0.035% 2-Mercaptoethanol and 100x B27 supplement with Retinoic Acid (RA). MEA recordings were performed on day 3, 5, 7, 9, 11, 13 at 37 °C in a Maestro MEA system with AxIS software using a bandwidth with a filter for 10Hz to 2.5 kHz cutoff frequencies. For the pharmacological experiment, 50 μM PYC-12 were applied to plate immediately before recording. For MEA recording, brain organoids treated with WSN were included as the control organoids. The phase contrast images of organoids seeded in the MEA plates were taken after MEA recording.

### Enzyme-linked immunosorbent assay (ELISA)

Inflammatory factors (TNF-α, INF-γ, IL-6, CCL2, COX2) in cultured supernatants of brain organoids with or without challenging by virus was measured using a commercial ELISA Kit (Dogesce). Briefly, samples were double diluted using the dilution buffer, and the optical density (OD) was measured at 450 nm with an ELISA reader (Beckman). The concentration of inflammatory factors was calculated according to the manufacturer's instruction.

### The whole transcriptome analysis

High throughput RNA sequencing was performed by Cloud-Seq Biotech (Shanghai, China). Total RNA was extracted from three organoids for each group (three biological replicates for each group) by TRIzol and the rRNAs were removed with NEBNext rRNA Depletion Kit (New England Biolabs, Inc., Massachusetts, USA). RNA libraries were constructed using the NEBNext® Ultra™ II Directional RNA Library Prep Kit (New England Biolabs, Inc., Massachusetts, USA) following the manufacturer's instructions. The libraries were quality-controlled and quantified using the BioAnalyzer 2100 system (Agilent Technologies, Inc., USA). The library sequencing was performed on an Illumina Hiseq instrument with 150 bp paired end reads. Paired-end reads were harvested from Illumina HiSeq 4000 sequencer, and quality-controlled by Q30. After 3' adaptor-trimming and removing low-quality reads by cutadapt software (v1.9.3), high-quality clean reads were aligned to the reference genome (UCSC MM10) with hisat2 software (v2.0.4). Guided by the Ensembl gtf gene annotation file, the cuffdiff software (part of cufflinks) was used to obtain the gene level FPKM as the expression profiles of mRNA. The total expressed gene number and LogFPKM of mRNA in different mouse groups were plotted and compared.

### Statistical analysis

All data were analyzed using the GraphPad Prism 9 software. For the statistical analysis of other results, statistical evaluation was performed by Student's unpaired t-test or one-way ANOVA with Tukey's multiple comparisons test. Data are presented as means ± SD or as described in the corresponding legends. A probability of p < 0.05 was considered as statistically significant. For annotations of significance, ^*^p < 0.05; ^**^p < 0.01; ^***^p < 0.001; ^****^p < 0.0001.

### Data availability

RNA sequencing data and report were uploaded to the figshare repository (DOI: 10.6084/m9.figshare.20051210). All other data supporting this study are available within this paper and its Supplementary Information.

## Results

### Generation and characterization of cerebral organoids from hPSCs

To study the neuropathogenesis of virus-infected brain organoids instead of patient-derived brain tissue, we first generated human pluripotent stem cell (hPSC)-derived brain organoids as previously described 39, with slight modifications (**Figure S1A**). The representative bright field images display the entire development of brain organoids at indicated time points (**Figure S1B**). Next, we performed immunostaining of brain organoids at days 15, 30, 60, and 120 with the neural stem cell marker-SOX2 and the neuron marker-MAP2 (**Figure S1C**). Statistical analysis showed that the majority of the cells on day 15 and day 30 organoids were neural stem cells (SOX2+), while the majority of the cells on day 60 and day 120 organoids were neurons (MAP2+) (**Figure S1D**). In summary, these results suggested that there was robust differentiation of brain organoids and following applications of virus infection model.

### Brain organoid as an *in vitro* model for distinct virus infection

**Figure 1:**
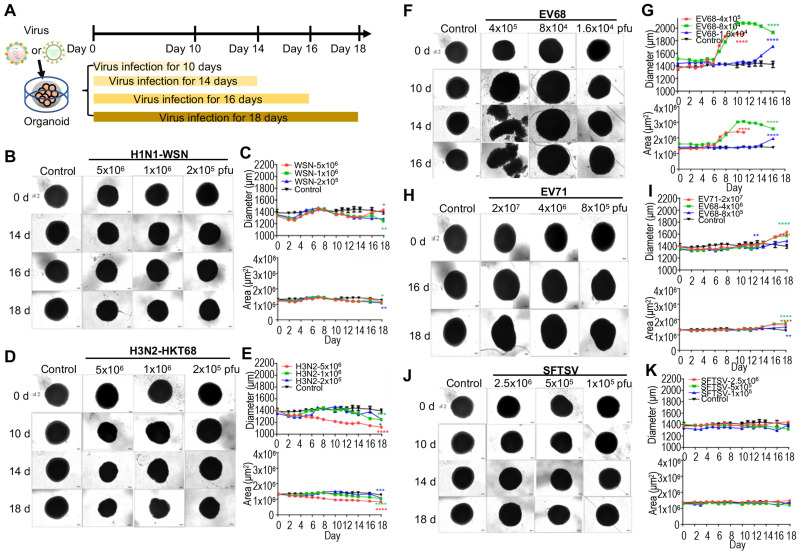
** Organoids as virus infection models.** (**A**) Schematic illustration of experimental design. Day 40 brain organoid was infected with viruses at indicated time. (**B, D, F, H, J**) Representative bright-field images of day 40-brain organoids infected with viruses at indicated time point, including H1N1-WSN, H3N2-HKT68, EV68, EV71 and SFTSV (n = 3 per group) (Note: The control group was the same in all groups). The infected concentrations for H1N1-WSN were 5x10^6^ pfu, 1x10^6^ pfu and 2x10^5^ pfu; for H3N2 were 5x10^6^ pfu, 1x10^6^ pfu and 2x10^6^ pfu; for EV68 were 4x10^5^ pfu, 8x10^4^ pfu and 1.6x10^4^ pfu; for EV71 were 2x10^7^ pfu, 4x10^6^ pfu and 8x10^5^ pfu. Scale bars, 50 μm. (**C, E, G, I, K**) Statistical analysis of area (μm^2^) and diameter (μm) of brain organoid infected with viruses at indicated time point. Measurement were collected till day 11 for the higher dose (red) of EV68 group due to the dissociation of organoid on day 14 and day 16. * p < 0.05, ** p < 0.01, *** p < 0.001, **** p < 0.0001.

To model the effects of virus infection on early human brain development, we used hPSC-derived brain organoids at day 40 of differentiation. According to previous studies, influenza virus 9, enterovirus 40 and severe fever with thrombocytopenia syndrome virus (SFTSV) 41 results in encephalitis. Therefore, we challenged the day 40 brain organoids with a diverse panel of viruses, including influenza viruses (H1N1-WSN and H3N2-HKT68), enteroviruses (EV68 and EV71) and SFTSV at indicated timepoints, and identified the optimal doses for virus infection according to our preliminary test. These were 1x10^6^ pfu for WSN, 5x10^6^ pfu for H3N2, 8x10^4^ pfu for EV68, and 4x10^6^ pfu for EV71 (**Figure 1A**). The representative bright field images showed a decrease in overall organoid size in the influenza virus infection group at indicated virus doses (**Figure 1B, D**), which was similar to Zika virus infection but not as severe 42. Statistical analysis of diameters (μm) and areas (μm^2^) of influenza virus-infected organoids showed a dose-dependent size decrease (**Figure 1C, E**). Meanwhile, enteroviruses (EV68 and EV71)-infected brain organoids resulted in a significant increase of overall organoid size at indicated virus doses through bright field images and statistical analysis of their diameters and areas (**Figure 1F-I**). However, SFTSV-infected brain organoids showed no obvious changes in overall organoid size (**Figure 1J, K**). Therefore, the morphological changes of organoids challenged by multiple viruses may be different due to different mechanisms.

**Figure 2:**
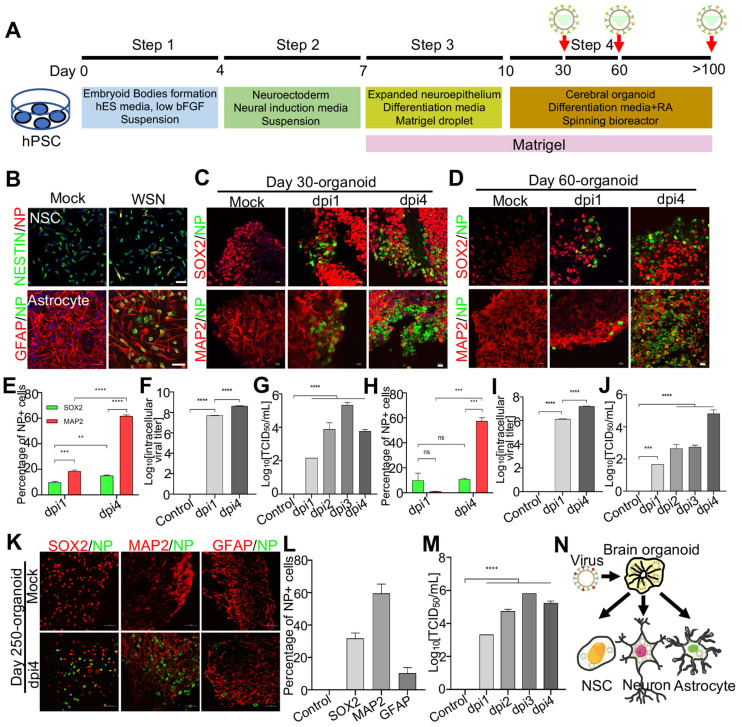
** Modeling Influenza virus *in vitro* using brain organoids.** (**A**) Schematic illustration of the experimental design of brain organoids infected with WSN at indicated time points. (**B**) Immunostaining of neural stem cells and astrocytes infected with WSN. Scale bars, 50 μm. (**C, D**) Immunostaining of neural stem cells and neurons of day 30 and day 60 brain organoids infected with WSN for 1 day and 4 days. Scale bars, 100 μm. (**E, H**) The percentage of NP+ cells in infected day 30 and day 60 brain organoid. ~10% of SOX2+ NSCs and ~60% of MAP2+ neurons were infected with WSN. (**F, G, I, J**) The viral titers of intracellular and supernatants on day 30 and day 60 brain organoids. (**K**) Immunostaining of neural stem cells, neurons and astrocytes of day 250 brain organoids infected with WSN for 1 day and 4 days. Scale bars, 100 μm. (**L**) The percentage of NP+ cells in infected brain organoid. ~30% of SOX2+ NSCs, ~60% of MAP2+ neurons and 10% astrocytes were infected with WSN. (**M**) The viral titers of supernatants on day 250 brain organoids. (**N**) Schematic illustration of WSN preferentially infected neurons in brain organoid. ** p < 0.01, *** p < 0.001, **** p < 0.0001.

Next, to investigate the mechanism under virus infection, we primarily focused on the study of WSN infected brain organoids. These brain organoids were generated as mentioned previously and infected on days 30, 60, and 250 (**Figure 2A**). Prior to WSN infected organoids, we first confirmed that ~20% NESTIN+ NSCs derived from hPSCs and ~10% GFAP+ astrocyte could be infected by WSN at the cellular level (**Figure 2B**). Then, we performed organoid infection at a multiplicity of infection (MOI) of 1. Immunostaining of day 30 and day 60 brain organoids with SOX2+ NSCs and MAP2+ neurons showed a time-dependent increase of nucleoprotein (NP)+ cells compared to mock infection, especially for MAP2+ neurons (**Figure 2C, D**). The statistical percentage of NP+ cells demonstrated that WSN was more likely to infect MAP2+ neurons (~60%) at 4 dpi, while only ~10% - 20% of NSCs were infected at 1 dpi and 4 dpi, respectively (**Figure 2E, H**). The intracellular and extracellular virus titers showed a time-dependent increase after WSN infection in day 30 or day 60 organoids (**Figure 2F, G, I, J**). To further investigate whether WSN could infect more mature organoids, we challenged day 250 brain organoid with WSN at aforementioned dose. Immunostaining of day 250 organoids revealed that ~30% of SOX2+ NSCs, ~60% of MAP2+ neurons, and ~10% of GFAP+ astrocytes were infected by WSN (**Figure 2K, L**). The extracellular virus titers showed a time-dependent increase of WSN infected day 250 organoids (**Figure 2M**). These results suggested that MAP2+ neurons were more susceptible to WSN infection compared to NSCs or astrocytes in brain organoids (**Figure 2N**).

### Transcriptomic profiling of WSN infected organoids

**Figure 3:**
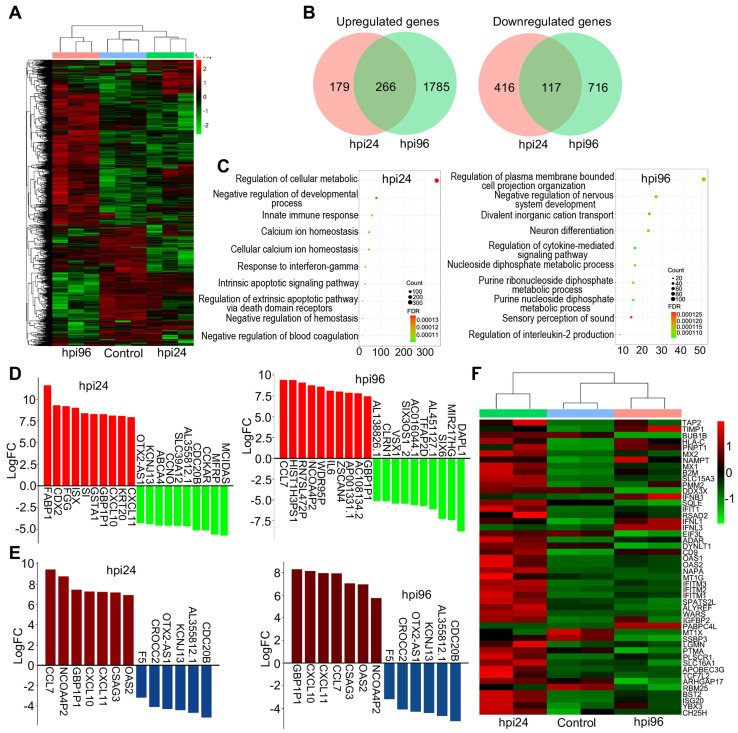
** The RNA transcriptomic analysis of human brain organoids after WSN infection.** (**A**) Day-60 organoid was infected with WSN at a multiplicity of infection (MOI) of 1 at 1 dpi and 4 dpi compared to mock-infected organoids (n=3). Hierarchical clustering heatmap of differentially expressed genes derived from the comparison among the group of control, hpi96 and hpi24. (**B**) Venn diagram of upregulated and downregulated genes of brain organoids infected with WSN at 1 dpi and 4 dpi. (**C**) Top 10 of enriched GO terms of brain organoids infected with WSN at 1 dpi and 4 dpi. (**D**) The top 10 of upregulated and downregulated genes at 1 dpi and 4 dpi after WSN infection. (**E**) The co-upregulated and co-downregulated genes at 1 dpi and 4 dpi after WSN infection. (**F**) Heatmap of interferon stimulating genes (ISGs).

To investigate the underlying molecular mechanisms at the gene level, we performed transcriptomic profiling of WSN-infected day-60 organoids at 1 dpi and 4 dpi compared to mock-infected organoids. Hierarchical clustering analysis showed the significant differences in gene expression between 1 dpi and 4 dpi organoids compared to mock-infected organoids (**Figure 3A**). A Venn diagram shows that there were more gene alterations at 4 dpi compared to 1 dpi, and 266 genes were upregulated while 117 genes were downregulated in both groups (**Figure 3B**). GO terms were mainly enriched in the regulation of cellular metabolic process, negative regulation of developmental process, and innate immune response at 1 dpi, while they were mainly enriched in negative regulation of nervous system development, and neuron differentiation at 4 dpi (**Figure 3C**). KEGG signaling pathway terms were enriched in virus infection pathways at 1 dpi, while they were enriched in MAPK signaling pathway and glycolysis/gluconeogenesis at 4 dpi (**Figure S2A**). Next, we performed a detailed gene expression analysis at 1 dpi and 4 dpi. In the top 10 of upregulated and downregulated genes, *FABP1*, *CDX2*, *FGG*, *ISX*, *SI*, *GSTA1*, *GBP1P1*, *CXCL10*, *KRT20*, and *CXCL11* were significantly upregulated, while *MCIDAS*, *MFRP*, *CCKAR*, *CDC20B*, *AL355812.1*, *SLC39A12*, *CCNO*, *ABCA4*, *KCNJ13*, and *OTX2-AS1* were significantly downregulated in the 1 dpi group. *CCL7*, *HIST1H3PS1*, *RN7SL472P*, *NCOA4P2*, *WDR95P*, *IL6*, *ZSCAN4*, *AP001331.1*, *AC108134.2*, and *GBP1P1* were significantly upregulated, while *DAPL1*, *MIR217HG*, *SIX6*, *AL451127.1*, *TFAP2D*, *AC016044.1*, *SIX3OS1_2*, *VSX1*, *CLRN1*, and *AL138826.1* were significantly downregulated in the 4 dpi group (**Figure 3D**). *GBP1P1, CXCL10, CXCL11, CCL7, CSAG3, OAS2*, and *NCOA4P2* were upregulated and *CDC20B*, *AL355812.1, KCNJ13, OTX2-AS1*, *CROCC2*, and *F5* were downregulated in both groups (**Figure 3E**). In most cells, interferon (IFN) response is a major first line of defense against viral infection 43. Viral infection triggers the production of IFNs, which then bind to ubiquitously expressed receptors on nearby cells and induce a powerful transcriptional program comprised hundreds of antiviral IFN-stimulated genes (ISGs) 44. In this study, hierarchical clustering analysis identified a set of ISGs (e.g., *IFITM1/2/3*, *BST2*, and *SLC16A1*) that were highly induced in the early stages of WSN infection (1 dpi), while the levels of interferons IFNB1, IFNL1 and IFNL2 are upregulated only in late stages of infection (4 dpi) (**Figure 3F**), indicating intracellular response to virus infection at different stage. We also analyzed some DEGs of transcription factors (**Figure S2B**), inflammatory factors (**Figure S2C**), and metabolic genes (**Figure S2D**) that were associated with virus infection. We found that they showed significant changes between 1 dpi and 4 dpi compared to mock infection. Moreover, a protein-protein interaction network of WSN infected brain organoids at 4 dpi was more robust than at 1 dpi (**Figure S2E**). In addition, WSN-infected organoids caused not only protein gene changes, but also some noncoding RNA levels, such as *BISPR* and *MIR4435-2HG* (**Figure S3**). Collectively, these results suggested that WSN infection of brain organoids resulted in considerable gene alterations, and implicating these genes for the development of new antiviral strategies.

### WSN impairs brain organoid growth through inducing apoptosis and inflammation

**Figure 4:**
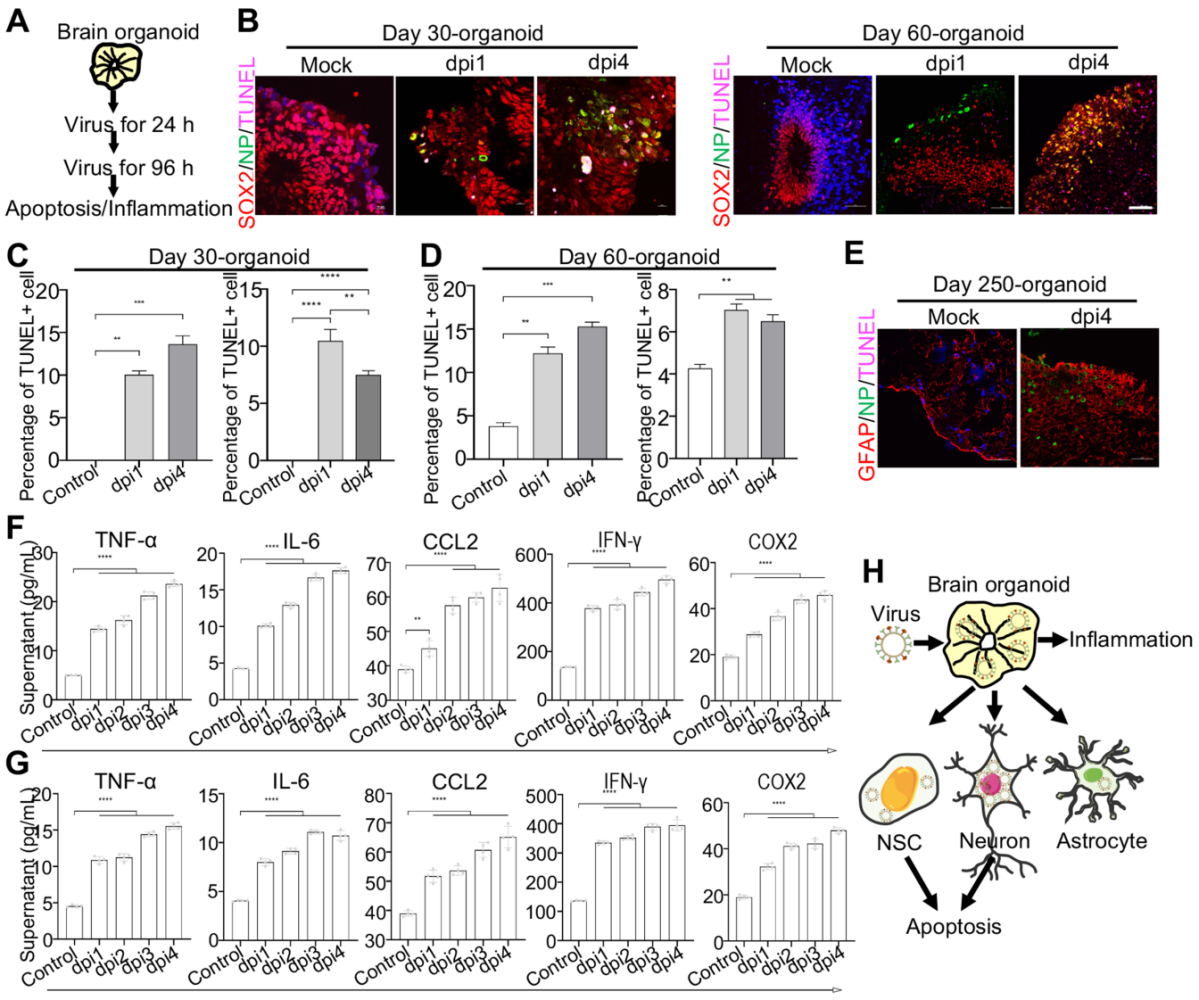
** WSN induced cell apoptosis and inflammation of human brain organoids.** (**A**) Schematic illustration of infection flow. Brain organoid was infected with WSN at a multiplicity of infection (MOI) of 1 at 1 dpi and 4 dpi compared to mock-infected organoids (n=3), then the apoptosis and inflammation were monitored. (**B-D**) The TUNEL staining and quantification of positive cells on day-30 and day-60 brain organoids infected with WSN at 1 dpi and 4 dpi in a time-dependent increase compared to control group, respectively (Left panel represents SOX2+ NSCs, right panel represents MAP2+ neurons in each figure of C-D). Scale bars, 10 μm. (**E**) The TUNEL staining of GFAP+ astrocytes of day 250 brain organoids at 4 dpi. (**F, G**) The secreted inflammatory factors (e.g., TNF-α, IL-6, CCL2, IFN-γ and COX2) of day-30 (**F**) and day-60 (**G**) brain organoids at indicated infection timepoint, respectively. (**H**) Summary of the impairment mechanism of WSN infected brain organoid. Scale bars, 100 μm. ** p < 0.01, *** p < 0.001, **** p < 0.0001.

To further investigate the neuropathogenesis of brain organoids subjected to WSN infection, we conducted related assays, including apoptosis and inflammatory factor release after WSN infection at 1 dpi and 4 dpi (**Figure 4A**). Significant cell apoptosis of SOX2+ NSCs in day 30 and day 60 brain organoids at 1 dpi and 4 dpi was observed by TUNEL staining (**Figure 4B**). The percentage of TUNEL+ cells was a time-dependent increase, and the apoptosis of NSCs was higher than neurons during early WSN infection (**Figure 4C, D**). Unlike Zika virus infection, which mainly induces the apoptosis of NSCs to result in microcephaly 42, WSN primarily infects MAP2+ neurons and causes the apoptosis of both NSCs and neurons. We also detected the apoptosis of GFAP+ astrocytes in day 250 organoids, and we did not observe obvious apoptosis (**Figure 4E**). Next, we determined the inflammatory factor levels in supernatants from days 30 and 60 brain organoids by enzyme linked immunosorbent assay (ELISA). We found that TNF-α, INF-γ, IL-6, CCL2, and COX2 in organoid supernatants were significantly increased in a time-dependent manner from days 30 and 60 brain organoids (**Figure 4F, G**). Taken together, these results suggested that WSN could impair brain organoid growth through eliciting apoptosis and inflammation (**Figure 4H**).

### Antiviral and neuroprotective effects of drugs and neurotrophic factors

**Figure 5:**
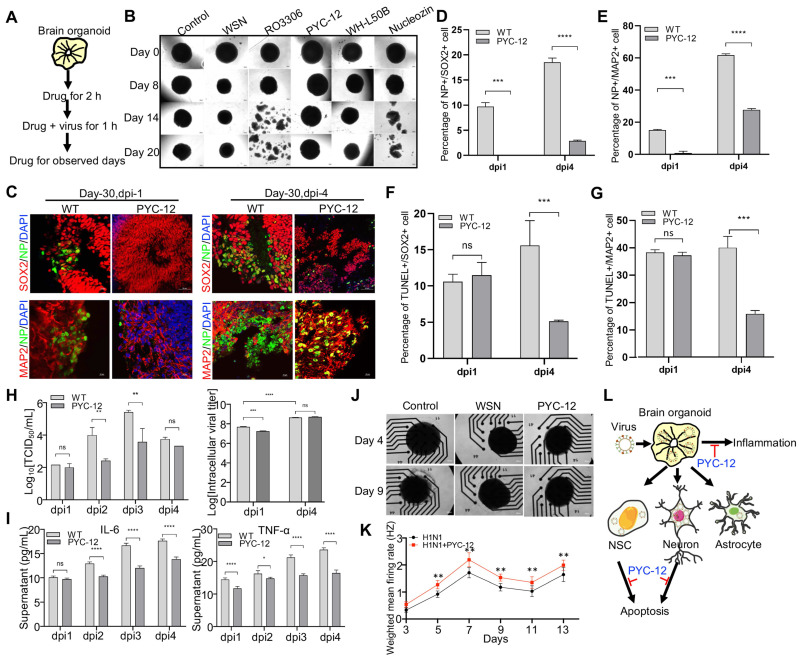
** Antiviral drug study of human brain organoids infected with influenza virus.** (**A**) Schematic of the workflow of drug screening. Brain organoids were first treated with compounds for 2 hours, followed by co-treatment with WSN and compounds for 1 hour, then continued to compounds treatment for observed days, respectively. (**B**) The representative bright field images of organoids cotreated with H1N1-WSN (MOI=1) and several drugs at indicated time points, respectively. Nucleozin was as positive control. Scale bars, 400 μm. (**C**) Immunostaining of neural stem cells and neurons of day 30 brain organoids co-treated with WSN and PYC-12 for 1 day and 4 days. Scale bars, 10 μm and 50 μm. (**D, E**) The statistical analysis of NP+ cells on day 30 brain organoids co-treated with WSN and PYC-12 for 1 day and 4 days. (**F, G**) The statistical analysis of TUNEL+ cells in neural stem cells and neurons of day 30 brain organoids co-treated with WSN and PYC-12 for 1 day and 4 days. Scale bars, 10 μm. (**H**) The viral titers of intracellular and supernatants on day 30 brain organoids co-treated with WSN and PYC-12. (**I**) The inflammatory factors release (IL-6 and TNF-α) of day-30 brain organoids co-treated with WSN and PYC-12 at indicated infection timepoint, respectively. (**J**) The representative bright field images of Microelectrode arrays (MEA) analysis of PYC-12 treated brain organoids. Scale bars, 50 μm. (**K**) Weighted mean firing rate (Hz) of brain organoids treated with H1N1 or cotreated with H1N1 and PYC-12 at indicated time points. (**L**) The schematic illustration of antiviral strategy of PYC-12 through anti-apoptosis and anti-inflammation. *p < 0.05, **p < 0.01, ***p < 0.001, ****p < 0.0001.

As these results prove, it turned out that WSN did impair brain organoids through inducing cell apoptosis and inflammation response. Therefore, we performed antiviral screening studies. We first analyzed several compounds, PYC-12 45, which has anti-inflammatory and antioxidant properties, RO3306 46, which is an ATP-competitive inhibitor, and WH-L50B, at the cellular level, and we used Nucleozin as a positive control, as it induces nuclear accumulation of influenza virus nucleoprotein (NP) leading to cessation of viral replication 47,48. Compared to RO3306, PYC-12, WH-L50B, and Nucleozin significantly suppressed virus infection (**Figure S4**). Next, we further evaluated the antiviral effect of four compounds at the organoid level. Briefly, day 40 brain organoids were first treated with four compounds for 2 hours, followed by co-treatment with WSN and compounds for 1 hour, then continued to compounds treatment for observed days, respectively (**Figure 5A**). In contrast to WSN infection group, PYC-12 could significantly rescue the morphological changes of brain organoids compared to the WSN infection group (**Figure 5B**). Besides, three compounds, peramivir, PYC-12, and WH-L50B, were used in drug screening of H3N2-infected organoids, and results showed that Peramivir, a positive drug, which is a highly selective inhibitor of influenza A and B neuraminidase 49, exhibited the highest antiviral effect compared to other compounds (**Figure S5**). Therefore, we used PYC-12 for our following studies. Immunostaining of SOX2+ NSCs and MAP2+ neurons on day 30 and day 60 brain organoids infected by WSN revealed that PYC-12 treatment could significantly inhibit WSN infection at 1 dpi and 4 dpi (**Figure 5C**). The statistical analysis of NP+ cells in both groups showed the similar results at 1 dpi and 4 dpi (**Figure 5D, E**). The statistical analysis of TUNEL+ cells in both groups showed that PYC-12 could significantly decrease the apoptosis of SOX2+ NSCs and MAP2+ neurons at 4 dpi (**Figure 5F, G**). PYC-12 also decreased the extracellular and intracellular viral titers (**Figure 5H**), as well as decreasing IL-6 and TNF-α production (**Figure 5I**). Microelectrode array (MEA) analysis showed that PYC-12 could increase the weighted mean firing rate (Hz) of brain organoids compared to WSN infection at indicated time points (**Figure 5J, K**). We primarily screened a potential drug candidate-PYC-12 against WSN infection based on our brain organoid models (**Figure 5L**).

In addition, neurotrophic factors (NFs), which are endogenous soluble proteins regulating the survival and growth of neurons, protect human normal brain functions against microbial pathogens have been reported in the literature 50. Thus, to investigate whether NFs also have antiviral effects in *in vitro* organoid models, we conducted antiviral assays with the same treatments. First, we demonstrated that brain-derived neurotrophic factor (BDNF), glial-derived neurotrophic factor (GDNF), neurotrophin-3 (NT3), and their combinations (GBN) could significantly inhibit WSN infection at the NESTIN+ NSCs level (**Figure S6A**), especially BDNF and GBN in NP+ cells (**Figure S6B**). All three NFs and GBN showed decreased intracellular virus titers (**Figure S6C**), while only GBN inhibited extracellular virus titers at the NSC level (**Figure S6D**). Then, we confirmed the antiviral effect of GBN on day 30 organoids (**Figure S6E, F**), and GBN could decrease intracellular and extracellular virus titers (**Figure S6G, H**). GBN significantly inhibited WSN infection through decreasing both cell apoptosis (**Figure S6I**) and inflammatory factor release (**Figure S6J**). At the gene level, we found that NF could significantly induce a set of ISG expressions, such as *MCL1*, *IFITM3*, *B2M*, *BST2*, *OAS1*, and *PKR* to function against virus infection (**Figure S7**). Collectively, neurotrophic factors also possessed antiviral roles via inducing ISG expression to suppress cell apoptosis and inflammation response in brain organoid models (**Figure S6K**), and implicating the possibility of a combination therapy in the future.

## Discussion

The neuropathogenesis of the human brain caused by influenza virus remains poorly understood. In the light of this fundamental problem, we designed an experimental platform using human brain organoids as a virus infection model to study this neuropathogenesis in detail. Brain organoids serve as an invaluable tool in the field of virus research to investigate the molecular events underlying virus replication and antiviral drug screening.

Previous study reported that Influenza A virus could infect neonatal mice in a wide range of brain regions potentially through the cerebrospinal fluid, and result in apoptotic cell death and gliosis in the areas of viral infection and inducing proinflammatory cytokine expression 28. And, Wang *et al.* revealed that Influenza virus induces inflammatory response in mouse primary cortical neurons with limited viral replication 51. Lee's team demonstrated that avian H7N9 and pandemic H1N1 viruses infected the differentiated human astrocytic (T98G) and neuronal (SH-SY5Y) cells, however, infectious progeny viruses can only be detected in H7N9 virus infected human neuronal cells, but not T98G cells 52. These findings, to some extent, provide some evidence for the infection tropism and inducing inflammatory response of influenza viruses at the level of human cell lines and mouse brain tissue, but do not fully represent the true state of influenza virus infected human brain. In our study, we first demonstrated that influenza virus (H1N1-WSN) could widely infect multiple cell types in brain organoids, including SOX2+ neural stem cells (NSCs), MAP2+ neurons, and GFAP+ astrocytes, and holds an infection tropism of MAP2+ neurons (**Figure 2**), which may support a direct link between influenza virus infection and the neurologic symptoms. Inflammatory factors (e.g., IL-6, TNF-α, and INF-γ) induced by WSN infection in brain organoids (**Figure 4F, G**) may be the major cause for viral entrance into the brain through damaging the blood brain barrier (BBB) 53. However, whether other virulent influenza viruses (e.g., H3N2, H5N1, or H7N9) have similar results needs further investigation in higher level of biosafety laboratories. Unlike WSN infection, Zika virus preferentially infects human cortical neural progenitor cells with high efficiency, but exhibits lower levels of infection in human embryonic stem cells (ESCs), hPSCs, and immature cortical neurons 43. Therefore, regional and cell type specific tropism of virus infection in brain organoid may directly support a link with specific pathological syndromes.

Although brain organoids can serve as a model of a three-dimensional organ to achieve multiple cellular interactions not possible with cell lines, organoids just partially model the early development characteristics of human tissues. Which type of neurons are specifically infected with influenza virus in brain organoids needs further study in our next work. Some research groups have leveraged virulent viruses (e.g., H5N1, H5N3, or H3N2) [54,55]to infect mice, and have demonstrated the route of brain infection, but cell type-specific tropism of viral infection in the central nervous system remain poorly understood. Therefore, if we completely confirm the cell type-specific infections of influenza or other viruses, it should provide a scientific basis for developing specific antiviral drugs. Single-cell RNA sequencing technology 56,57 could be used to track specific cell types infected by viruses in space and time through detecting the gene expression of viruses, while human brain tissue infected by viruses are very hard to get and labeling hundreds or thousands of nerve cells is difficult for current single-cell RNA sequencing technology.

In addition, we found that WSN could infect astrocytes (**Figure 2K, L**), but not induce their apoptosis (**Figure 4E**), which was similar to brain organoids infected with SARS-CoV-2 58. This finding may indicate that virus-infected astrocytes can lead to peripheral cell death by creating a locally hypoxic and a resource-restricted environment for cells. Furthermore, we performed whole RNA transcriptomic analysis for comparison of a control and 4 dpi and 1 dpi group and identified some upregulated protein targets (e.g., CCL7, NCOA4P2, GBP1P1, CXCL10, CXCL11, CSAG3, and OAS2) and noncoding RNAs (e.g., BISPR, AC116407.2, AC092687.3, and MIR4435-2HG), implicating these for the development of new antiviral targets. However, whether these targets can exert antiviral effects needs to be further validated in future work. Finally, we performed antiviral screening using brain organoids and found a potential antiviral drug, PYC-12, which could significantly suppress virus replication, apoptosis of NSCs and neurons, and inflammatory responses, and restore electrophysiological function (**Figure 5**). Additionally, excitotoxicity has been implicated in the pathogenesis of virus-induced diseases of the CNS in animal model 59-64. Few studies reported excitotoxicity induced by influenza virus in human brain. In our study, we demonstrated that WSN infection decreased electrophysiological function (**Figure 5J, K**), which may indicate that WSN could induce excitotoxicity. Therefore, further investigation, such as excitotoxicity induced by influenza virus infection, will be conducted in our future work. Besides, we also explored the antiviral and neuroprotective effects of neurotrophic factors such as GDNF, BDNF, and NF3 (**Figure S6**), and demonstrated that they also did suppress WSN infection, the apoptosis of NSCs and neurons, and inflammatory responses. This work also highlights the possibility of a combinations therapy of drugs and neurotrophic factors in clinical treatment.

## Conclusion

In this study, using an *in vitro* brain organoid model, we demonstrated that MAP2+ neurons were more susceptible to influenza virus (H1N1-WSN) infection, unveiled the neuropathogenesis of the brain through inducing apoptosis and inflammation, and conducted antiviral screening to achieve antiviral and neuroprotective aims. In summary, we establish a tractable experimental model system to investigate the impact and mechanism of influenza virus on human brain development, and provide a platform for identifying therapeutic compounds.

## Supplementary Material

Supplementary figures and tables.

## References

[1] Tregoning JS, Schwarze J (2010). Respiratory viral infections in infants: Causes, clinical symptoms, virology, and immunology. Clin Microbiol Rev.

[2] Keipp Talbot H, Falsey AR (2010). The diagnosis of viral respiratory disease in older adults. Clin Infect Dis.

[3] Englund J, Feuchtinger T, Ljungman P (2011). Viral infections in immunocompromised patients. Biol Blood Marrow Transplant.

[4] Vehapoglu A, Turel O, Uygur Sahin T, Kutlu NO, Iscan A (2015). Clinical significance of human metapneumovirus in refractory status epilepticus and encephalitis: case report and review of the literature. Case Rep Neurol Med.

[5] Hoekstra PJ (2019). Attention-deficit/hyperactivity disorder: is there a connection with the immune system?. Eur Child Adolesc Psychiatry.

[6] Sauer AK, Stanton JE, Hans S, Grabrucker AM (2021). Autism spectrum disorders: etiology and pathology. Autism Spectr Disord.

[7] Koyuncu OO, Hogue IB, Enquist LW (2013). Virus infections in the nervous system. Cell Host Microbe.

[8] Johansson A, Mohamed MS, Moulin TC, Schi¨oth HB (2020). Neurological manifestations of COVID-19 : A comprehensive literature review and discussion of mechanisms. J Neuroimmunol.

[9] McGavern DB, Kang SS (2011). Illuminating viral infections in the nervous system. Nat Rev Immunol.

[10] Han J, Perez J, Schafer A (2018). Influenza virus: small molecule therapeutics and mechanisms of antiviral resistance. Curr Med Chem.

[11] Liang CY, Yang CH, Lin JN (2018). Focal encephalitis, meningitis, and acute respiratory distress syndrome associated with influenza A infection. Med Princ Pract.

[12] Qian X, Song H, Ming G (2019). Brain organoids: advances, applications and challenges. Development.

[13] De Crignis E, Hossain T, Romal S (2021). Application of human liver organoids as a patient-derived primary model for HBV infection and related hepatocellular carcinoma. Elife.

[14] Cao Y (2022). The uses of 3D human brain organoids for neurotoxicity evaluations: A review. Neurotoxicology.

[15] Chhibber T, Sounak Bagchi, Lahooti B (2020). CNS organoids: an innovative tool for neurological disease modeling and drug neurotoxicity screening. Drug Discov Today.

[16] Chen X, Sun G, Tian E (2021). Modeling sporadic alzheimer's disease in human brain organoids under serum exposure. Adv Sci.

[17] Dang J, Tiwari SK, Lichinchi G (2016). Zika virus depletes neural progenitors in human cerebral organoids through activation of the innate immune receptor TLR3. Cell Stem Cell.

[18] Garcez PP, Loiola EC, Da Costa RM (2016). Zika virus: Zika virus impairs growth in human neurospheres and brain organoids. Science.

[19] Depla JA, Mulder LA, de Sá RV (2022). Human brain organoids as models for central nervous system viral infection. Viruses.

[20] Sun G, Chiuppesi F, Chen X (2020). Modeling human cytomegalovirus-induced microcephaly in human iPSC-derived brain organoids. Cell Reports Med.

[21] Ramezankhani R, Solhi R (2022). Organoid and microfluidics-based platforms for drug screening in COVID-19. Drug Discov Today.

[22] Zhou Z, Cong L, Cong X (2021). Patient-derived organoids in precision medicine: drug screening, organoid-on-a-chip and living organoid biobank. Front Oncol.

[23] Sun N, Meng X, Liu Y, Song D, Jiang C, Cai J (2021). Applications of brain organoids in neurodevelopment and neurological diseases. J Biomed Sci.

[24] Qian L, Julia TCW (2021). Human iPSC-based modeling of central nerve system disorders for drug discovery. Int J Mol Sci.

[25] Yu J (2021). Organoids: A new model for SARS-CoV-2 translational research. Int J Stem Cells.

[26] Song E, Zhang C, Israelow B (2021). Neuroinvasion of SARS-CoV-2 in human and mouse brain. J Exp Med.

[27] Baltagi SA, Shoykhet M, Felmet K, Kochanek PM, Bell MJ (2010). Neurological sequelae of 2009 influenza A (H1N1) in children: A case series observed during a pandemic. Pediatr Crit Care Med.

[28] Yu JE, Kim M, Lee JH, Chang BJ, Song CS, Nahm SS (2014). Neonatal influenza infection causes pathological changes in the mouse brain. Vet Res.

[29] Shi L, Fatemi SH, Sidwell RW, Patterson PH (2003). Maternal influenza infection causes marked behavioral and pharmacological changes in the offspring. J Neurosci.

[30] Glaser CA, Winter K, DuBray K (2012). A population-based study of neurologic manifestations of severe influenza A(H1N1)pdm09 in california. Clin Infect Dis.

[31] Rohn TT, Catlin LW (2011). Immunolocalization of influenza a virus and markers of inflammation in the human Parkinson's disease brain. PLoS One.

[32] Jang H, Boltz D, Sturm-Ramirez K (2009). Highly pathogenic H5N1 influenza virus can enter the central nervous system and induce neuroinflammation and neurodegeneration. Proc Natl Acad Sci U S A.

[33] Plourde JR, Pyles JA, Layton RC, Vaughan SE, Tipper JL, Harrod KS (2012). Neurovirulence of H5N1 infection in ferrets Is mediated by multifocal replication in distinct permissive neuronal cell regions. PLoS One.

[34] Kim JH, Yu JE, Chang BJ, Nahm SS (2018). Neonatal influenza virus infection affects myelination in influenza-recovered mouse brain. J Vet Sci.

[35] Aronsson F, Lannebo C, Paucar M, Brask J, Kristensson K, Karlsson H (2002). Persistence of viral RNA in the brain of offspring to mice infected with influenza A/WSN/33 virus during pregnancy. J Neurovirol.

[36] Rubin SA, Liu D, Pletnikov M (2004). Wild-type and attenuated influenza virus infection of the neonatal rat brain. J Neurovirol.

[37] Silvano FD, Yoshikawa M, Shimada A, Otsuki K, Umemura T (1997). Enhanced neuropathogenicity of avian influenza A virus by passages through air sac and brain of chicks. J Vet Med Sci.

[38] Lippmann ES, Estevez-Silva MC, Ashton RS (2014). Defined human pluripotent stem cell culture enables highly efficient neuroepithelium derivation without small molecule inhibitors. Stem Cells.

[39] Lancaster MA, Knoblich JA (2014). Generation of cerebral organoids from human pluripotent stem cells. Nat Protoc.

[40] Chen BS, Lee HC, Lee KM, Gong YN, Shih SR (2020). Enterovirus and encephalitis. Front Microbiol.

[41] Cao J, Lu G, Wen L (2021). Severe fever with thrombocytopenia syndrome virus (SFTSV)-host interactome screen identifies viral nucleoprotein-associated host factors as potential antiviral targets. Comput Struct Biotechnol J.

[42] Tang H, Hammack C, Ogden SC (2016). Zika virus infects human cortical neural progenitors and attenuates their growth. Cell Stem Cell.

[43] Wu X, Dao Thi VL, Huang Y (2018). Intrinsic immunity shapes viral resistance of stem cells. Cell.

[44] Malterer MB, Glass SJ, Newman JP (2014). Interferon-stimulated genes: A complex web of host defenses. Annu Rev Immunol.

[45] Canali R, Comitato R, Schonlau F, Virgili F (2009). The anti-inflammatory pharmacology of Pycnogenol® in humans involves COX-2 and 5-LOX mRNA expression in leukocytes. Int Immunopharmacol.

[46] Lebrec V, Poteau M, Morretton JP, Gavet O (2022). Chk1 dynamics in G2 phase upon replication stress predict daughter cell outcome. Dev Cell.

[47] Kao RY, Yang D, Lau LS (2010). Identification of influenza A nucleoprotein as an antiviral target. Nat Biotechnol.

[48] Gerritz SW, Cianci C, Kim S (2011). Inhibition of influenza virus replication via small molecules that induce the formation of higher-order nucleoprotein oligomers. Proc Natl Acad Sci U S A.

[49] Kohno S, Kida H, Mizuguchi M (2011). Intravenous peramivir for treatment of influenza A and B virus infection in high-risk patients. Antimicrob Agents Chemother.

[50] Platholi J, Lee FS (2018). Chapter 5 - Neurotrophic factors. Second Edi. Handbook of Developmental Neurotoxicology (Second Edition). Elsevier Inc.

[51] Wang G, Li R, Jiang Z (2016). Influenza virus induces inflammatory response in mouse primary cortical neurons with limited viral replication. Biomed Res Int.

[52] Ng YP, Yip TF, Peiris JSM, Ip NY, Lee SMY (2018). Avian influenza A H7N9 virus infects human astrocytes and neuronal cells and induces inflammatory immune responses. J Neurovirol.

[53] Shlosberg D, Benifla M, Kaufer D, Friedman A (2010). Blood-brain barrier breakdown as a therapeutic target in traumatic brain injury. Nat Rev Neurol.

[54] Mori I, Kimura Y (2001). Neuropathogenesis of influenza virus infection in mice. Microbes Infect.

[55] Sadasivan S, Zanin M, O'Brien K, Schultz-Cherry S, Smeyne RJ (2015). Induction of microglia activation after infection with the non-neurotropic A/CA/04/2009 H1N1 influenza virus. PLoS One.

[56] Hwang B, Lee JH, Bang D (2018). Single-cell RNA sequencing technologies and bioinformatics pipelines. Exp Mol Med.

[57] Ofengeim D, Giagtzoglou N, Huh D, Zou C, Yuan J (2017). Single-cell RNA sequencing: unraveling the brain one cell at a time. Trends Mol Med.

[58] Song E, Zhang C, Israelow B (2020). Neuroinvasive potential of SARS-CoV-2 revealed in a human brain organoid model Eric. bioRxiv.

[59] Andersson T, Schultzberg M, Schwarcz R, Love A, Wickman C, Kristensson K (1991). NMDA-receptor antagonist prevents measles virus-induced neurodegeneration. Eur J Neurosci.

[60] Darman J, Backovic S, Dike S (2004). Viral-induced spinal motor neuron death is non-cell-autonomous and involves glutamate excitotoxicity. J Neurosci.

[61] Lee JM, Zipfel GJ, Choi DW (1999). The changing landscape of ischaemic brain injury mechanisms. Nature.

[62] Nargi-Aizenman JL, Griffin DE (2001). Sindbis virus-induced neuronal death Is both necrotic and apoptotic and is ameliorated by N-methyl-d-aspartate receptor antagonists. J Virol.

[63] Nargi-Aizenman JL, Havert MB, Zhang M, Irani DN, Rothstein JD, Griffin DE (2004). Glutamate receptor antagonists protect from virus-induced neural degeneration. Ann Neurol.

[64] Weli SC, Scott CA, Ward CA, Jackson AC (2006). Rabies virus infection of primary neuronal cultures and adult mice: railure to demonstrate evidence of excitotoxicity. J Virol.

