# Supplementary material for: Recapitulating influenza virus infection and facilitating antiviral and neuroprotective screening in tractable brain organoids
Source: Theranostics. 2022 Jul 7;12(12):5317–29. doi: 10.7150/thno.75123 (PMC9330524; doi:10.7150/thno.75123)
Supplement: Supplementary file 1 — Supplementary figures and tables. [file thnov12p5317s1.pdf]

Supplementary material

Recapitulating influenza virus infection and facilitating antiviral and neuroprotective screening in tractable brain organoids

Xiaodong Zhang[1†], Haishuang Lin[1,2†✉], Liangzhen Dong[1†], Qing Xia[1✉]

[1]Department of Chemical Biology, State Key Laboratory of Natural and Biomimetic Drugs, School of Pharmaceutical Sciences, Peking University, Beijing, China.

[2]Institute of Laboratory Animal Science, Chinese Academy of Medical Sciences and Comparative Medicine Center, Peking Union Medical College, Beijing, China.

[†]These authors contributed equally to this work.

[✉]Corresponding: hlin9@pku.edu.cn; xqing@hsc.pku.edu.cn.

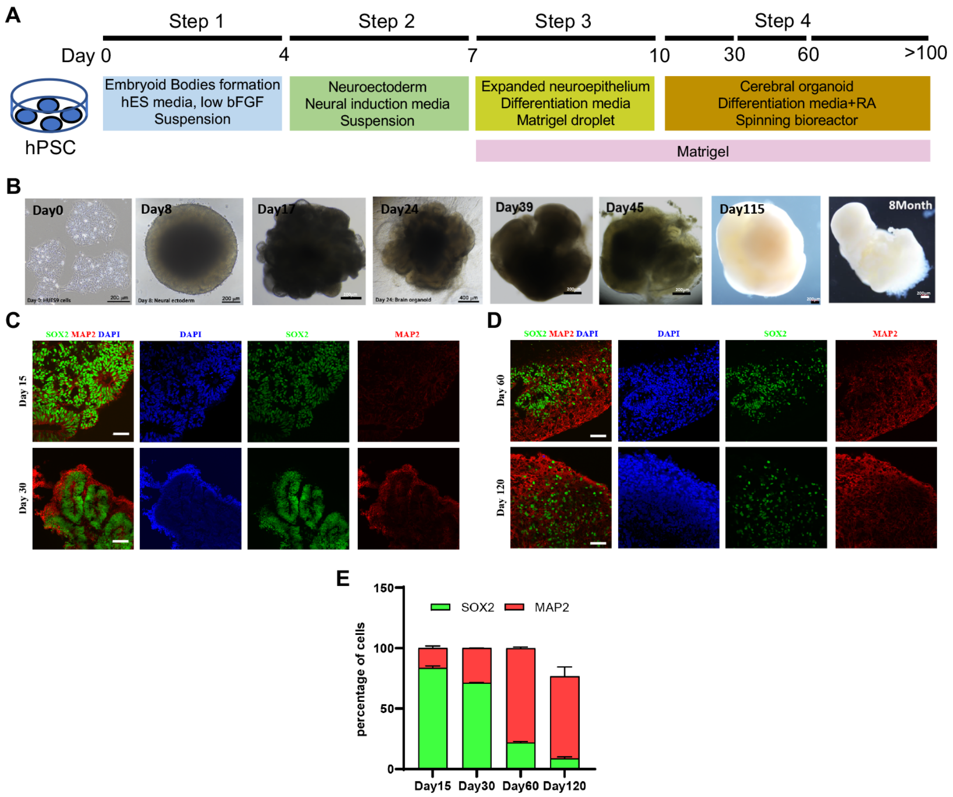

Figure S1. Generation and characterization of brain organoids. (**A**) Schematic illustration of the organoids generation. (**B**) Representative bright-field images of brain organoid derived from hPSCs at day 0, day 8, day 17, day 24, day 39, day 45, day 115 and month 8 brain organoids. Scale bars, 50 μm, 200 μm and 400 μm. (**C, D**) Immunostaining of brain organoids with SOX2+ NSCs and MAP2+ neurons on day 15, 30, 60 and 120. Scale bars, 50 μm and 100 μm. (**E**) The percentage of SOX2+ and MAP2+ cells on day 15-, 30-, 60-, and 120-brain organoid. The majority of cells on day 15- and 30-organoid were SOX2+ neural stem cells and MAP2+ neurons on day 60- and 120-organoid (n = 3).

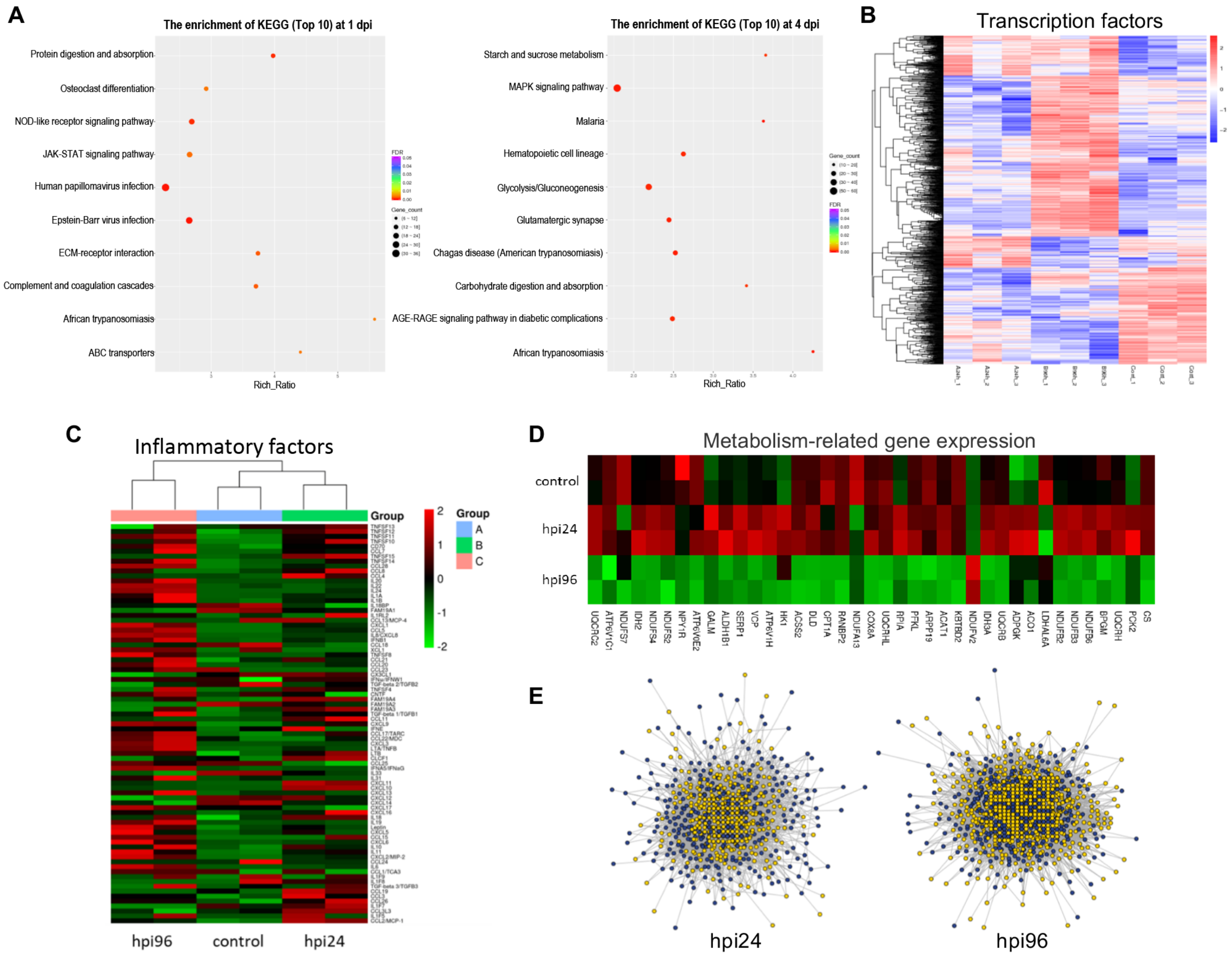

Figure S2. (**A**) The top 10 of KEGG pathway enrichment of of brain organoids infected with WSN at 1 dpi and 4 dpi. (**B**) Heatmap of transcriptional factors expression among control, hpi24 and hpi96. (**C**) Heatmap of inflammatory factors expression among control, hpi24 and hpi96. (**D**) Heatmap of metabolic genes. (**E**) Protein-protein interaction network of WSN infected brain organoids at 1 dpi and 4 dpi.

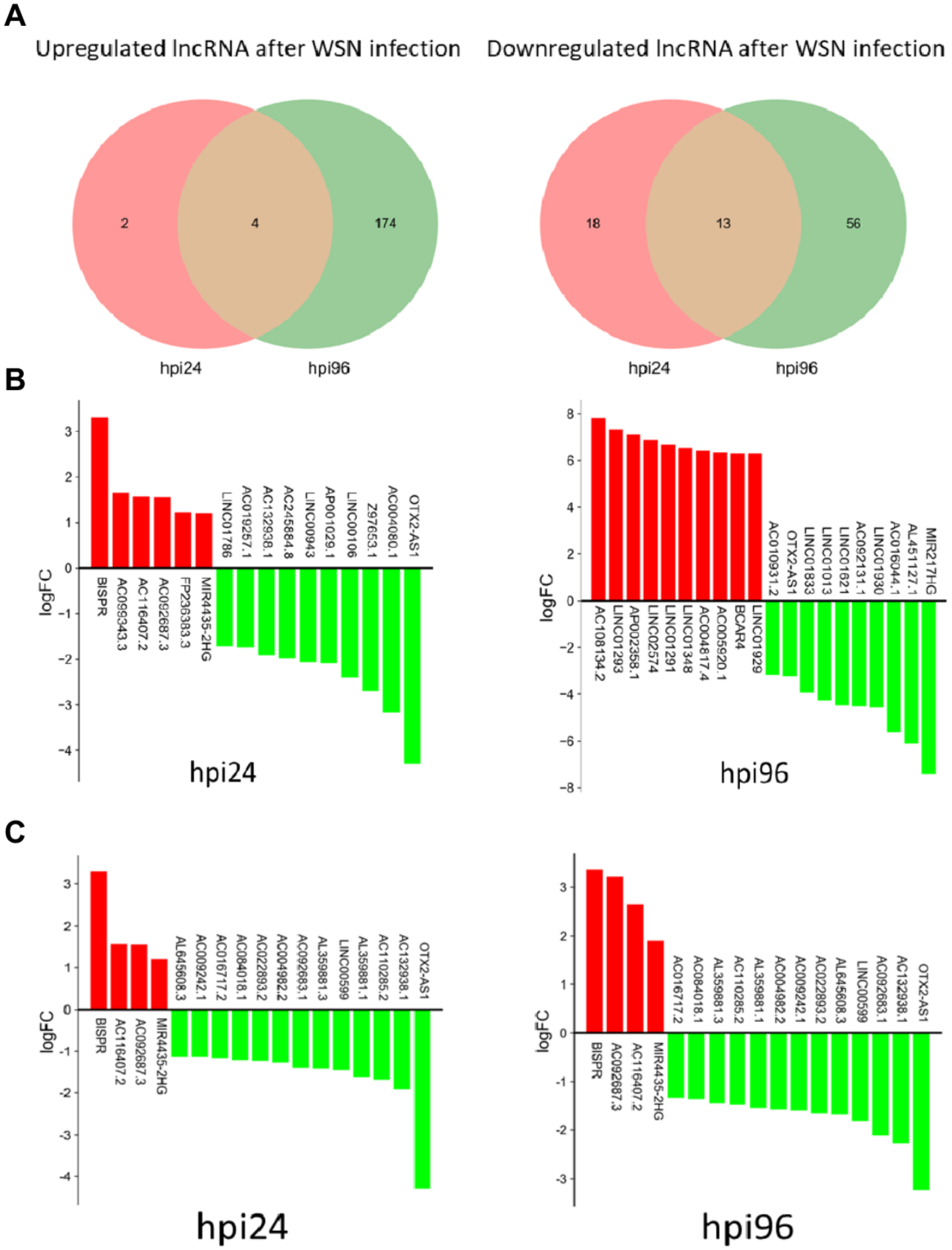

Figure S3. (**A**) Venn diagram of upregulated and downregulated lncRNAs of brain organoids infected with WSN at 1 dpi and 4 dpi. (**B**) The top of upregulated and downregulated lncRNAs at 1 dpi and 4 dpi after WSN infection. (**C**) The co-upregulated and co-downregulated lncRNAs at 1 dpi and 4 dpi after WSN infection.

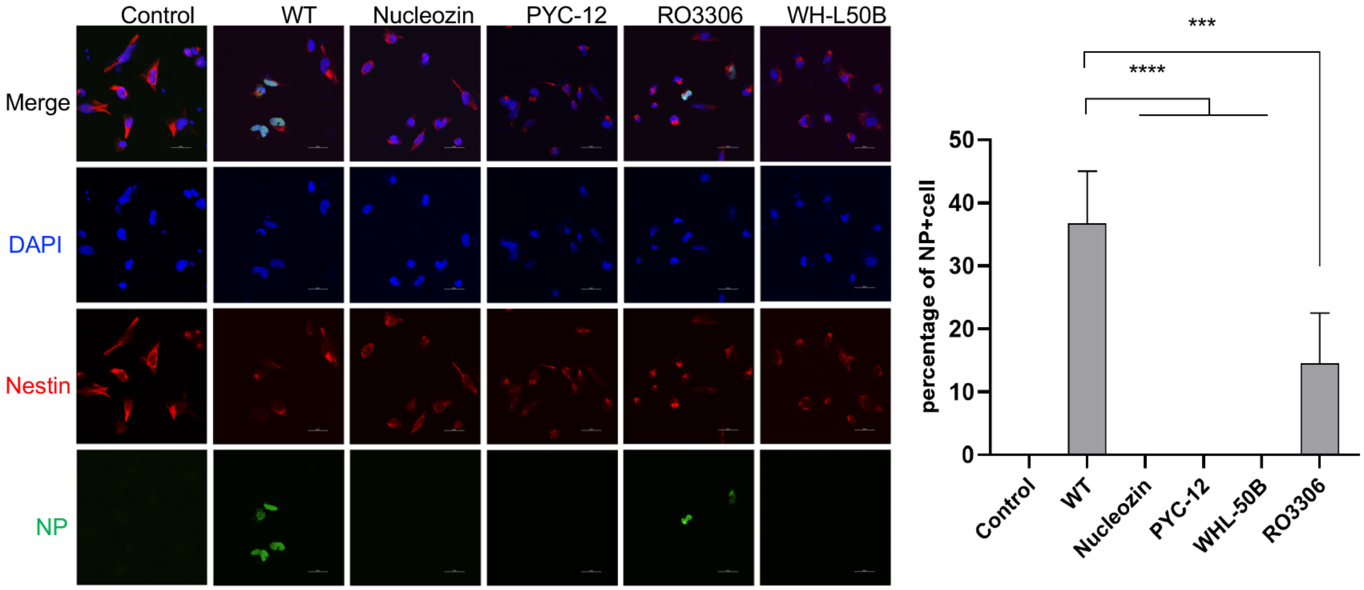

Figure S4. Antiviral drug study of NSCs. Human pluripotent stem cells derived neural stem cells were first treated with four compounds for 2 hours, followed by co-treatment with WSN and compounds for 1 hour, then continued to compounds treatment for observed days, respectively. Immunostaining and statistical analysis of neural stem cells treated with WSN, Nucleozin, PYC-12, RO3306 and WH-L50B. Non-treated NSCs was as a negative control. Scale bars, 20 μm. *** $p<0.001$, **** $p<0.0001$.

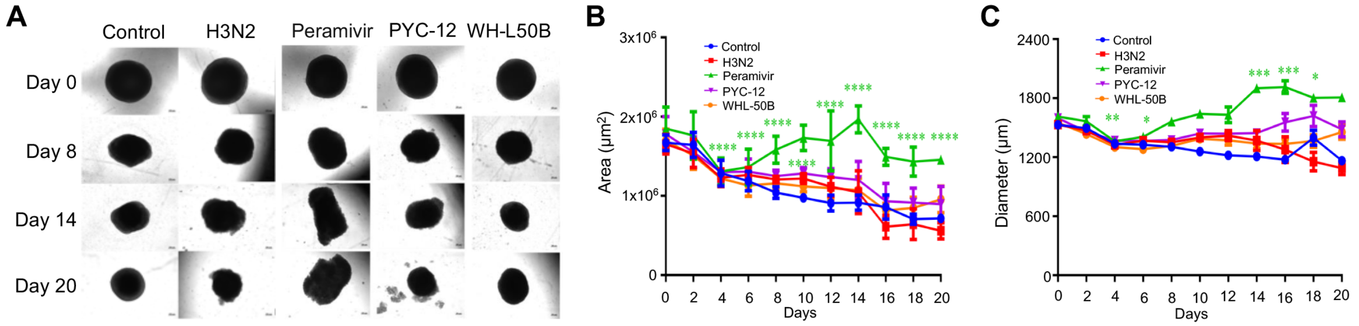

Figure S5. Antiviral drug study of human brain organoids infected with influenza virus. Brain organoids were first treated with compounds for 2 hours, followed by co-treatment with WSN and compounds for 1 hour, then continued to compounds treatment for observed days, respectively. (**A**) The phase images of brain organoids cotreated with H3N2-HKT68 and Peramivir, PYC-12 and WH-L50B at indicated time points, respectively. Scale bars, 50 μm. (**B, C**) Statistical analysis of area (μm^2) and diameter (μm) of organoids cotreated with H3N2-HKT68 and different drugs at indicated time points, respectively. * $p<0.05$, ** $p<0.01$, ***$p<0.001$, ****$p<0.0001$.

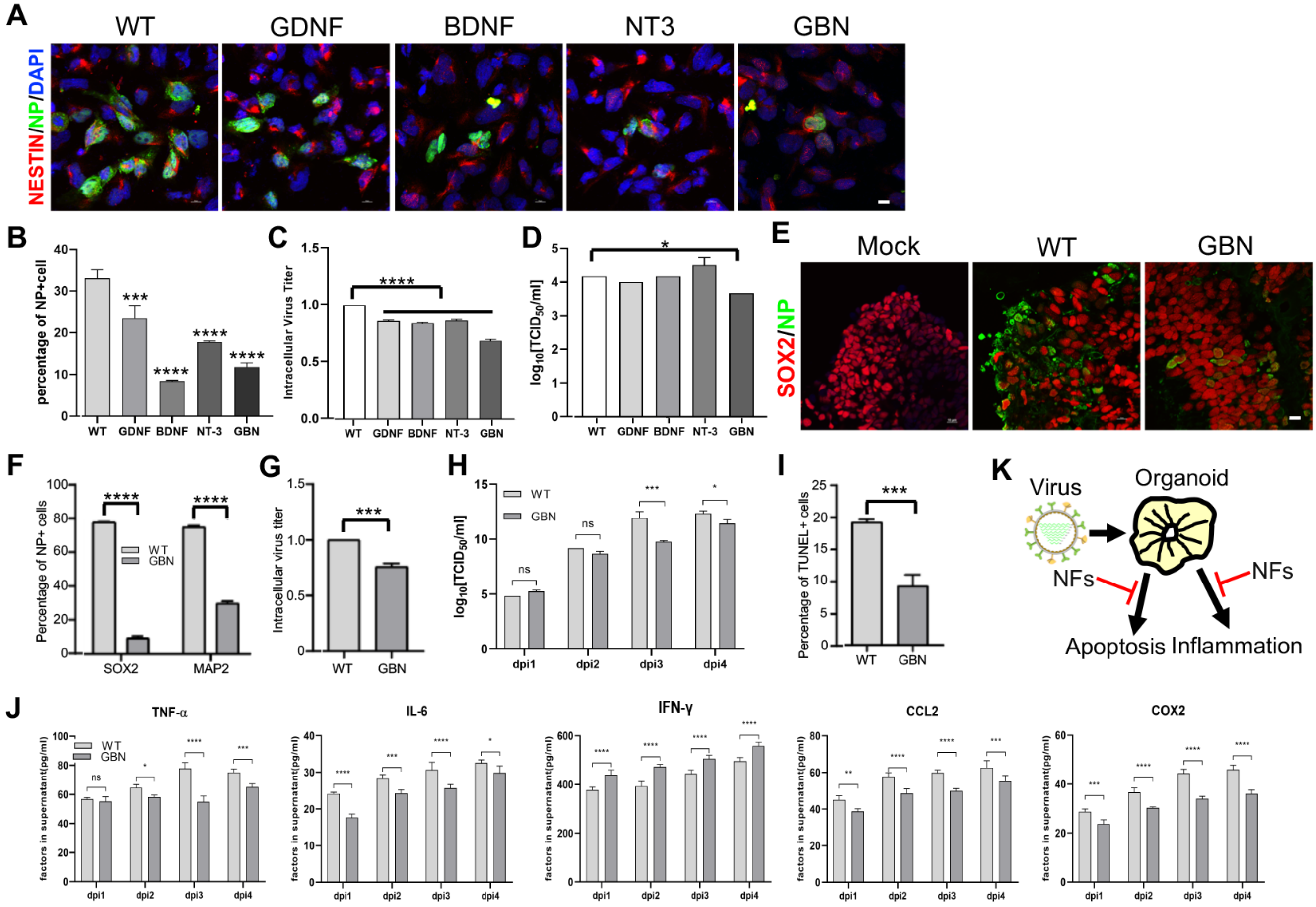

Figure S6. Neurotrophic factors inhibited WSN infection. Brain organoids were first treated with compounds for 2 hours, followed by co-treatment with WSN and neurotrophic factors for 1 hour, then continued to compounds treatment for observed days, respectively. (**A**) Immunostaining of NSCs treated with WSN or different neurotrophic factors, including BDNF, GDNF and NT3. GBN indicated the combination of these three neurotrophic factors. Scale bar, 10 μm. (**B**) The percentage of NP+ cells after treatments. (**C, D**) Intracellular (left) and extracellular (right) virus titers after treatments. (**E**) Immunostaining of day 30 brain organoid treated with WSN and GBN. Scale bar, 10 μm. (**F**) The percentage of NP+ cells after GBN treatment compared to WSN infection. (**G, H**) The intracellular and extracellular virus titers after treatments. (**I**) The TUNEL staining and quantification of positive cells on day-30 brain organoids at 4 dpi. Scale bar, 20 μm. (**J**) The secreted inflammatory factors (e.g., TNF-α, IL-6, CCL2, IFN-γ and COX2) of day-30 brain organoids at indicated infection timepoints, respectively. (**K**) Schematic illustration of antiviral methods through neurotrophic factors treatments. * $p<0.05$, ** $p<0.01$, *** $p<0.001$, **** $p<0.0001$.

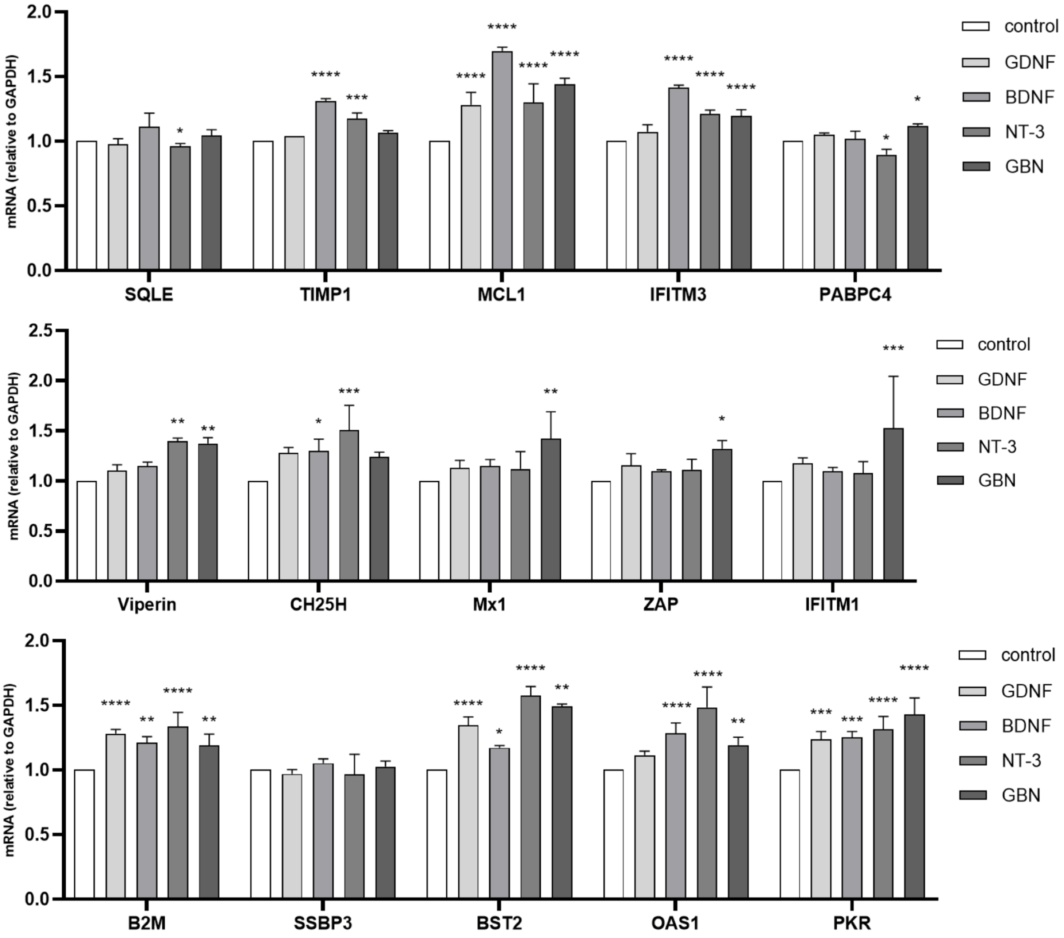

Figure S7. Insulin stimulating genes (ISGs) expression treated with neurotrophic factors was monitored by quantitative RT-PCR. * $p<0.05$, ** $p<0.01$, *** $p<0.001$, **** $p<0.0001$.

Table S1. Antibodies used in this study.

Antibody	Supplier	Catalog. No	Species	Dilution
SOX2	Abcam	ab171380	Mouse	1:200 (IF&FC)
MAP2	Abcam	ab32454	Rabbit	1:200 (IF&FC)
NP	Sino Biological	11675-MM03T	Mouse	1:200 (IF&FC)
GFAP	Abcam	ab4648	Mouse	1:200 (IF&FC)
NESTIN	Abcam	ab105389	Rabbit	1:200 (IF&FC)
Caspase 3	Abcam	ab32351	Rabbit	1:1000 (IF&FC)
TUNEL	ThermoFisher	C10617		
Secondary antibody	Abcam	Alexa Fluor-488	Rabbit	1:500
Secondary antibody	Abcam	Alexa Fluor-555	Rabbit	1:500
Secondary antibody	Abcam	711-165-152	Mouse	1:500
Secondary antibody	Abcam	705-605-147	Mouse	1:500

Table S2. Real time qPCR primers used in this study.

**Primer Name**	**Forward 5'-3'**	**Reverse 5'-3'**
M1	TGTGTAGGAAGCTTAAGAGGG	GTGGATTGGTTATTATCACC
IFITM3	TCCACCGTGATCCACATC	GATGTTCAGGCACTTGGC
SQLE	TGCTCTCCTACCGCTGTC	GATGTACAGGCAGCTGTTC
MCL1	TCATGTCGCCCGAAGAGG	CTCGTCCTCCTCCTCCTC
PABPC4	TCCTGCAAGGTGGTGTGT	CGAGACTTGAATCTGCCCAC
PAX3	TGTGCCCAGGATGATGCG	CATCTCCACGATCTTGTGGC
MX1	GTTACCAGGACTACGAGATTGAG	GGATGTACTTCTTGATGAGTGTCTT
IFITM1	GCACCATCCTTCCAAGGT	CATCTTCCTGTCCCTAGACTTC
CH25H	TACAAGATCCACCCTGACTTCT	AAGAGTAGCAGGCAGAACAG
OAS1	CCTCAGTCCTCTCACCACT	TCAACTGACCCAGGGCAT
PKR	TGCACGCAGATAATCACGG	TACTCGCTGTCTGTCAACC
ZAP	GTCCGAGCGGAATTTATGC	GTGGTCACAGATGTGGAGT
Viperin	TCTCGCTATCTCCTGTGACAG	GAACACTTTCCAGCGGACAG
BST2	TGATGGAGTGTCGCAATGT	GTGATCTCTCCCTCAAGCTC
SSBP3	TTATGTCACCGCGATACGC	GCATTGATCCTCCCATGTTG
B2M	TCTTTCTGGCCTGGAGGC	CATGGTTCACACGGCAGG
GAPDH	TCGACAGTCAGCCGCATCTTCTTT	ACCAAATCCGTTGACTCCGACCTT
β-actin	CAATGTGGCCGAGGACTTTG	CATTCTCCTTAGAGAGAAGTGG
